# Aquaculture mediates global transmission of a viral pathogen to wild salmon

**DOI:** 10.1126/sciadv.abe2592

**Published:** 2021-05-26

**Authors:** Gideon J. Mordecai, Kristina M. Miller, Arthur L. Bass, Andrew W. Bateman, Amy K. Teffer, Jessica M. Caleta, Emiliano Di Cicco, Angela D. Schulze, Karia H. Kaukinen, Shaorong Li, Amy Tabata, Brad R. Jones, Tobi J. Ming, Jeffrey B. Joy

**Affiliations:** 1 Department of Medicine, University of British Columbia, Vancouver, BC, Canada.; 2 Pacific Biological Station, Fisheries and Oceans Canada, Nanaimo, BC, Canada.; 3 Department of Forest and Conservation Sciences, Forest Sciences Centre, 3041 2424 Main Mall, Vancouver, BC V6T 1Z4, Canada.; 4 Department of Forest and Conservation Sciences, University of British Columbia, Vancouver, BC, Canada.; 5 Pacific Salmon Foundation, 1682 W 7th Ave., Vancouver, BC V6J 4S6, Canada.; 6 Department of Ecology and Evolutionary Biology, University of Toronto, 25 Willcocks Street, Room 3055, Toronto, ON M5S 3B2, Canada.; 7 Salmon Coast Field Station General Delivery, Simoom Sound, BC V0P 1S0, Canada.; 8 David H. Smith Conservation Research Fellowship, Society for Conservation Biology, Washington, DC, USA.; 9 Department of Microbiology and Immunology, University of British Columbia, Vancouver, BC, Canada.; 10 BC Centre for Excellence in HIV/AIDS, Vancouver, BC, Canada.; 11 Bioinformatics Programme, University of British Columbia, Vancouver, BC, Canada.

## Abstract

Global expansion of aquaculture and agriculture facilitates disease emergence and catalyzes transmission to sympatric wildlife populations. The health of wild salmon stocks critically concerns Indigenous peoples, commercial and recreational fishers, and the general public. Despite potential impact of viral pathogens such as Piscine orthoreovirus-1 (PRV-1) on endangered wild salmon populations, their epidemiology in wild fish populations remains obscure, as does the role of aquaculture in global and local spread. Our phylogeographic analyses of PRV-1 suggest that development of Atlantic salmon aquaculture facilitated spread from Europe to the North and South East Pacific. Phylogenetic analysis and reverse transcription polymerase chain reaction surveillance further illuminate the circumstances of emergence of PRV-1 in the North East Pacific and provide strong evidence for Atlantic salmon aquaculture as a source of infection in wild Pacific salmon. PRV-1 is now an important infectious agent in critically endangered wild Pacific salmon populations, fueled by aquacultural transmission.

## Teaser

Molecular epidemiology reveals that aquaculture contributes to emergence of a fish virus at both global and local epidemic hierarchies.

## INTRODUCTION

Pacific salmon (*Oncorhynchus* spp.) are foundation species, essential for transporting nutrients and energy between aquatic and terrestrial environments (*1*). However, there have been widespread declines of native Chinook (*O. tshawytscha*), sockeye (*O. nerka*), and coho (*O. kisutch)* salmon, with some populations extirpated and others on the brink of extinction (*2*–*5*). Resulting effects on the ecosystem are many. In particular, diminishing Chinook salmon populations contribute to declines in endangered Southern resident killer whales (*Orcinus orca*) (*6*). The stagnation or decline in wild fishery landings (tied to increasing demand) (*7*) has resulted in the rapid growth of aquaculture (which itself relies on forage fish from wild fisheries) (*8*). The history of disease emergence in aquaculture closely parallels the emergence of infectious disease in agricultural settings (*9*–*13*), and international trade has facilitated the global emergence and spread of infectious diseases (*14*). An unambiguous example is the appearance of diverse infectious agents of salmon after the rapid growth of salmon farming in Chile (home to no native salmonids) (*14*). Consequently, the risk of disease transmission from farmed to wild fish has increased (*15*), with potential to contribute to declines in wild fish populations, but the probability and magnitude of this transmission has not been determined.

Historical introductions of Atlantic salmon to the Pacific coast of North America (*16*) began in 1874 (*17*) and proceeded for a century in a failed attempt to develop naturalized populations. Egg imports were considerably bolstered with the expansion of aquaculture from the 1980s until imports ceased in 2009 (*18*). Similarity of emerging infectious agents in the Pacific Ocean with those associated with Atlantic salmon in Europe (*19*, *20*) suggests that these importations may be a source of transmission. However, the specifics of any given introduction often remain unclear. For instance, *Piscine orthoreovirus* (PRV) was first characterized in 2010 (*21*) and has been the subject of controversy for its potential to spread from salmon farms to wild salmon populations, putting wild fish at risk (*22*).

The source and age of PRV in the North East (NE) Pacific is contentious (*23*, *24*), with very low-load putative detections (unverified by sequencing) as long ago as 1977 (*25*). These detections are considered putative findings only, and to validate them, a peer-reviewed study would need to sequence archival PRV from 1977 and should include sufficient controls to screen out contaminants. The temporal signal in PRV-1 sequence data (*26*) enables phylogenetic validation of archival sequences, which would be expected to be phylogenetically basal to more recent PRV-1 sequences in the NE Pacific. The earliest confirmed PRV detection comes from a Chinook salmon sampled in 1992 (*25*). A recent publication concluded that PRV-1 originates from the North Atlantic but found that estimates of the timing of the introduction varied depending on the dataset used, either solely the S1 segment or full genomes (*26*).

Presently, PRV comprises three strains, PRV-1, PRV-2, and PRV-3 (*27*). Through infection with purified virus, all three strains of PRV have been identified as causative agents of disease (*27*–*29*). Phylogenetic analysis of the S1 and M2 viral gene segment groups PRV-1 into two main monophyletic clades (*30*, *31*), referred to as substrains PRV-1a and PRV-1b. PRV disease pathways (and associated clinical signs) are known to differ among virus strains and host species.

Various hypotheses have been proposed for the emergence and global spread of PRV-1. First, Siah *et al.* (*23*) suggested that PRV-1a may have originated from the Pacific [although the same author recently disproved this hypothesis (*26*)]. Second, PRV-1 is hypothesized to originate in Europe (*31*), where the development of aquaculture in Norway then facilitated divergence of PRV-1 into two substrains (*30*). It is argued (*30*) that this increase in viral genetic diversity occurred via reassortment of genomic segments from an unknown donor, resulting in emergence of PRV-1b and heart and skeletal muscle inflammation (HSMI) disease. To date, there is no evidence of a “donor strain,” and PRV-1a has also been associated with HSMI (*32*, *33*). Alternatively, incomplete lineage sorting may explain why evolutionary divergence is observed on some, but not all, viral genome segments. In this third scenario, when viral lineages separated, genomic divergence would have occurred in a selection of segments and not others, with the result that some segments remain similar in both PRV-1 subpopulations after the divide.

There are apparent differences in virulence between isolates of PRV-1 infecting Atlantic salmon (*34*). Recent work found that all PRV-1 isolates tested have the potential to cause moderate and (in some cases) severe pathological changes, but the severity of the disease varies between isolates (*35*). The same study has shown that it is not the S1 and M2 segments alone that result in differences in virulence, and the authors suggest that virulence may be linked to combined involvement and possible linkage of several genomic segments.

Virulence of viral strains in one host species cannot be extrapolated to virulence (or pathogenicity) of the same strain in different host species (*36*). Mounting evidence suggests that PRV-1a is associated with jaundice/anemia in Chinook salmon, despite reports of an absence of clinical disease in laboratory challenges with PRV-1a in Chinook (which in some cases may be the result of experimental design) (*37*, *38*).

Modest to moderate pathological lesions have been documented in PRV-1a–challenged Chinook salmon (*34*, *37*), and lesions consistent with these have been associated with PRV-1a infection in the NE Pacific in fish dying with jaundice and anemia on Chinook salmon farms (*33*) and in wild juvenile Chinook (*39*). Rather than the cardiac disease caused by PRV-1 in Atlantic salmon (*28*), PRV-1–associated lesions in Chinook salmon seem to affect primarily the kidney and liver (*33*), a manifestation consistent with other PRV-related diseases described in Pacific salmonids (*27*, *29*, *40*, *41*) and Atlantic salmon in eastern Canada (*42*).

In the NE Pacific, the same lineage, PRV-1a, is found in wild and farmed salmon (*33*), raising questions of the magnitude and direction of viral transmission between farmed and wild populations and—of critical importance for fisheries management—whether transmission poses a risk to wild fish. In addition, in the NE Pacific, salmon enhancement hatcheries subsidize natural salmon populations, and since 1950, 3.7 billion Chinook salmon have been released into the Salish Sea from hatcheries in the United States and British Columbia (BC) (*43*). The risk posed from emerging infectious disease associated with hatchery fish varies (*13*, *44*, *45*) and is largely unknown with respect to PRV-1.

Analysis of viral genomes can help determine the evolutionary history of infectious agents and quantify transmission dynamics between populations (*46*). In this study, we more than double the number of available full PRV-1 genomes and use epidemiological and phylogenetic methods to test hypotheses concerning the global spread of PRV-1 as well as transmission of PRV-1 between farmed Atlantic and wild salmon in the NE Pacific.

## RESULTS AND DISCUSSION

### Origin and global spread of PRV-1

To infer the evolutionary and transmission history of PRV-1, we combined all publicly available sequences, the oldest from 1988 with 86 newly sequenced genomes from the NE Pacific (fig. S1). We explored the temporal signal in the sequence data using root-to-tip regression, revealing that PRV-1a in the NE Pacific is evolving in a clocklike manner, with genetic diversity accruing proportional to time (fig. S2).

**Fig. 1:**
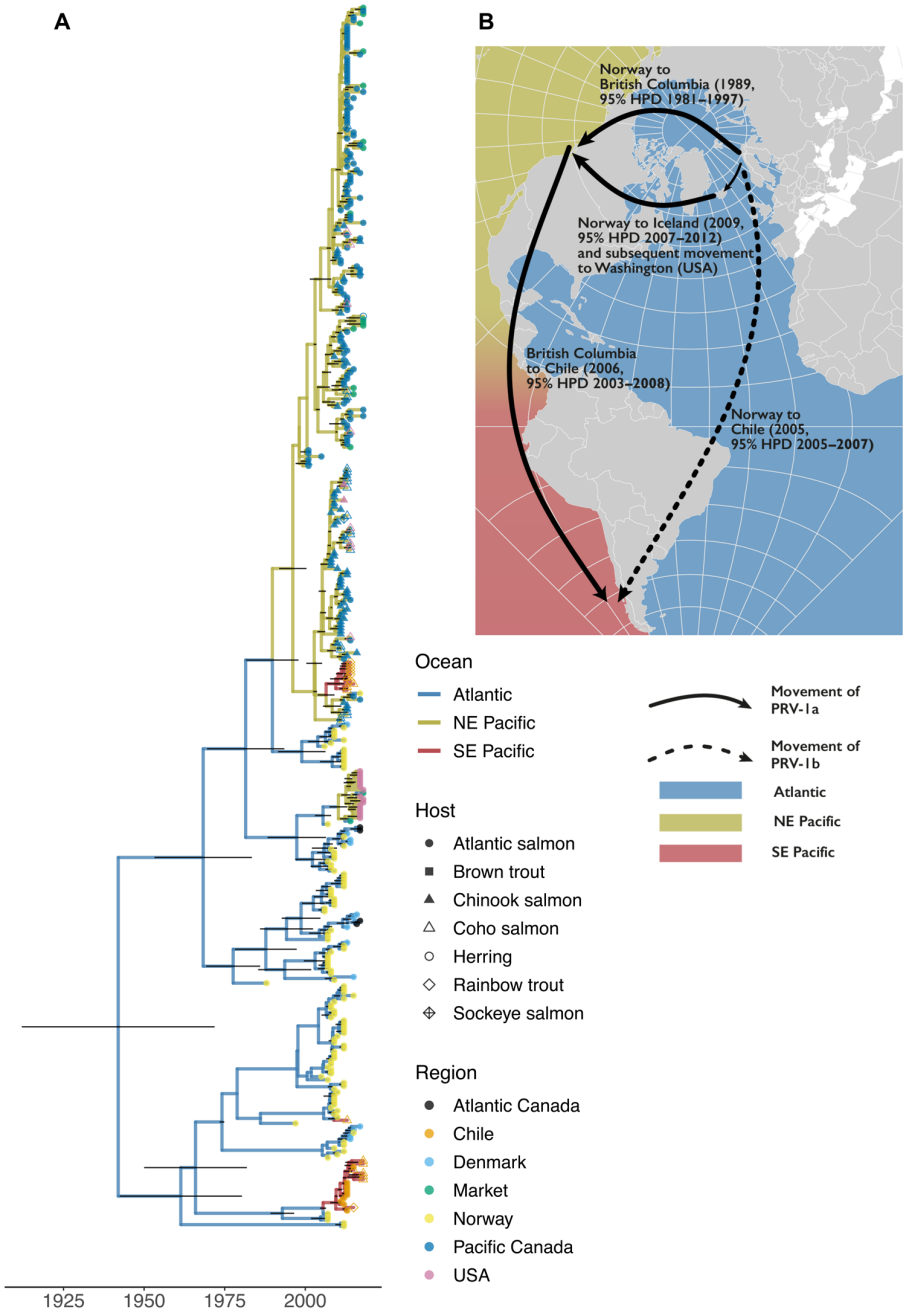
Global transmission of PRV-1. (**A**) Maximum clade credibility time-scaled phylogenetic tree based on the S1 genome segments (*n* = 270) and full genome sequences (*n* = 122) where available. Branches are colored by reconstruction of the origin of PRV-1. Samples were classed as Atlantic (Europe and Atlantic Canada), NE Pacific (Pacific Canada and USA), or South East Pacific (i.e., Chile). Vertical bars show the 95% HPD of the age estimate. Branch tips are colored and shaped by the species of the host and region in which the samples were collected. As the origin of samples collected from markets cannot always be determined, we assigned these to their own group. (**B**) Schematic representation of the global emergence of PRV-1. Arrows depict estimated translocations of PRV lineages. Movements are determined by the tree in (A).

Estimated divergence of PRV-1a and PRV-1b in 1942 [95% highest posterior density (HPD) interval, 1908–1968; Fig. 1 and table S1] precedes the 1970 advent of marine net-pen salmon aquaculture in Norway (*47*), suggesting that both substrains were either a natural part of wild Atlantic salmon populations or they diverged during the development of trout farming in Norway (*47*). However, reassortment of the S1 and M2 segments with an unknown donor virus could have occurred more recently, and our estimate cannot account for this. PRV-1b is associated with diseased farmed fish in Norway, and we predict that aquaculture-mediated selection may maintain PRV-1b at high frequency in Norwegian farms, while the less virulent (*30*) [but still pathogenic (*32*)] PRV-1a substrain is more common in wild fish in the Atlantic (*15*, *48*).

We estimate that PRV-1a in the NE Pacific diverged from PRV-1a in the Atlantic Ocean in 1989 (95% HPD, 1981–1997), and this suggests a recent introduction of PRV-1 to the NE Pacific (Fig. 1). This is consistent with the timing of Atlantic salmon egg imports from Europe for salmon farms in the NE Pacific. The estimate predates the first report, in 2002 (*32*), of a PRV-related disease (cardiomyopathy) in the NE Pacific. Introductions of Atlantic salmon to the Pacific (largely derived from the Eastern US) began more than a century ago, with the aim of establishing a self-recruiting fishery (*17*), but our estimate of the arrival of PRV-1 is much later. It is not clear why PRV-1 was not present in earlier introductions. We hypothesize that PRV-1 was not as common in the past as it is now (due to its association with aquaculture), so the chance of it being introduced before the development of aquaculture may have been lower. Alternatively, if a lineage of PRV-1 had been introduced, then we propose that this lineage has since undergone extinction in the Pacific and is no longer circulating today.

Although the majority of PRV-1 sequences in the NE Pacific appear to be descendants of this introduction, a later establishment [likely from Icelandic eggs (*49*)] is apparent in distinct PRV-1a sequences from escaped Atlantic salmon in Washington state (Fig. 1 and table S1).

By comparison, the presence of PRV-1b in Chile results in a greater diversity of PRV-1 in the South East Pacific than in the NE Pacific. Our analyses suggest two separate introductions of different PRV-1 substrains into Chile (Fig. 1), predating the first PRV-related diseases reported in Chile (*50*). Our analyses suggest, first, that PRV-1b was introduced into Chile directly from the Atlantic circa 2005 (95% HPD, 2005–2007) and, second, that PRV-1a was introduced to Chile via the NE Pacific in 2006 (95% HPD, 2003–2008), perhaps through Pacific salmon aquacultural egg translocations from the NE Pacific, which began in the 1970s and ceased in 2007 (*14*).

### Epidemiological role of aquaculture in PRV-1 transmission

**Fig. 2:**
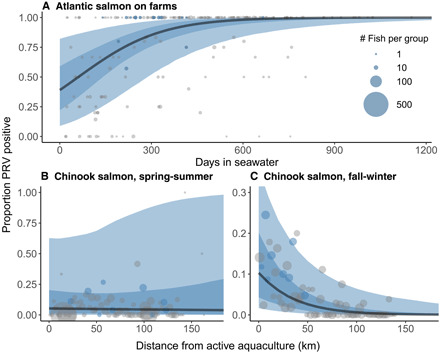
PRV-1 infection dynamics in farmed Atlantic and wild Chinook salmon. (**A**) Proportion of PRV-1–positive BC Atlantic salmon aquaculture audit samples (*n* = 664) versus time in saltwater. Points show the prevalence in each farm sample, scaled by sample size. Points colored blue contained a sample that was sequenced for phylogenetic analysis. The black curve is the fit of a Bayesian mixed-effects logistic model; almost all individuals on a farm become infected over the course of an 18-month grow-out period. Dark blue shading shows 95% credible interval (CI) of the mean logistic trend, and light blue shading shows 95% credible region including hierarchical variability from sampling events, farms, and aquaculture management zones. (**B** and **C**) The probability of PRV infection for first–marine-year Chinook salmon increases closer to active salmon farms in the fall-winter period, but not in the spring-summer. Chinook salmon (*n* = 4979) were captured by trawl and seine from 2008 through 2018 in coastal BC. Points (scaled by sample size) indicate the proportion of Chinook that were positive for PRV at varying distances from active aquaculture (2-km bins). Points colored blue contained a sample that was sequenced for phylogenetic analysis. The black curve is the fit of a Bayesian mixed-effects logistic model with random effects for regional population groupings and ocean entry year. Dark blue shading shows 95% CI for mean trends, and light blue shading shows 95% credible region, including hierarchical variation from ocean entry year and population grouping.

To test the hypothesis that PRV-1 commonly infects farmed salmon in BC, we examined viral infection dynamics in Atlantic (Fig. 2A) and Pacific (fig. S3) salmon farms. Prevalence of PRV-1 increased over an Atlantic salmon farm cohort’s production cycle, from ocean entry to harvest, approximately 18 months later (Fig. 2A). Across all Atlantic salmon farms, estimated PRV prevalence reached 97.2% [95% credible interval (CI): 80.6 to 99.8%] after 18 months, suggesting that Atlantic salmon farms are potentially a source of PRV to the surrounding environment. Detections of PRV were also common in farmed Pacific salmon (fig. S3A), but the relationship with time was not as evident as that in Atlantic farms. This could, in part, be due to fewer samples being available from Pacific salmon farms, especially in the first 6 months of ocean residence, but different scenarios are possible: Hatchery-reared fish may enter the farms infected (at uncertain and possibly variable prevalence), or they may enter the ocean uninfected and become infected over time. Alternatively, infection rates may remain the same or even decline over time. We do not have enough data to support any given scenario, and further research with a more frequent sampling regime is needed to determine the circumstances of PRV-1 infection in Chinook farms.

To indirectly assess transmission from farmed to wild salmon, we investigated the probability of PRV infection for wild Chinook salmon in relation to distance to active aquaculture facilities. In the fall and winter, Chinook salmon in the study area primarily originate from rivers in BC, and for these fish, PRV-1 infection was closely tied to farm proximity (Fig. 2C). Prevalence was near zero in BC fish collected over 100 km from active aquaculture, although we did not account for other factors that might influence PRV-1 prevalence, for instance, different environmental conditions or differences in host condition between regions. In agreement with previous studies (*22*, *51*, *52*), this correlation implicates transmission of PRV-1 from farmed Atlantic salmon to wild salmon. During fall and winter, molecular and histopathological signatures of PRV-related disease occur in both farmed (*33*) and wild (*39*) Chinook salmon from BC.

By contrast, there was no strong pattern of spatial correlation with proximity to active aquaculture in the spring and summer (Fig. 2B), when PRV-positive Chinook primarily came from Columbia River stocks migrating north of the west coast of Vancouver Island. Mostly absent in the region during fall and winter (*53*), these fish displayed a higher PRV-1 infection rate than Chinook from BC rivers (fig. S4). Of PRV-positive fish from the Columbia River, 85% were fin-clipped (hatchery marked), suggesting that hatcheries may play a role in the maintenance of PRV in the Columbia River system. Furthermore, many Columbia River fish carried a distinct lineage of PRV-1a. As these stream-type Columbia River Chinook rapidly migrate northward past Vancouver Island (*54*), they have limited contact with farms within coastal inlets.

**Fig. 3:**
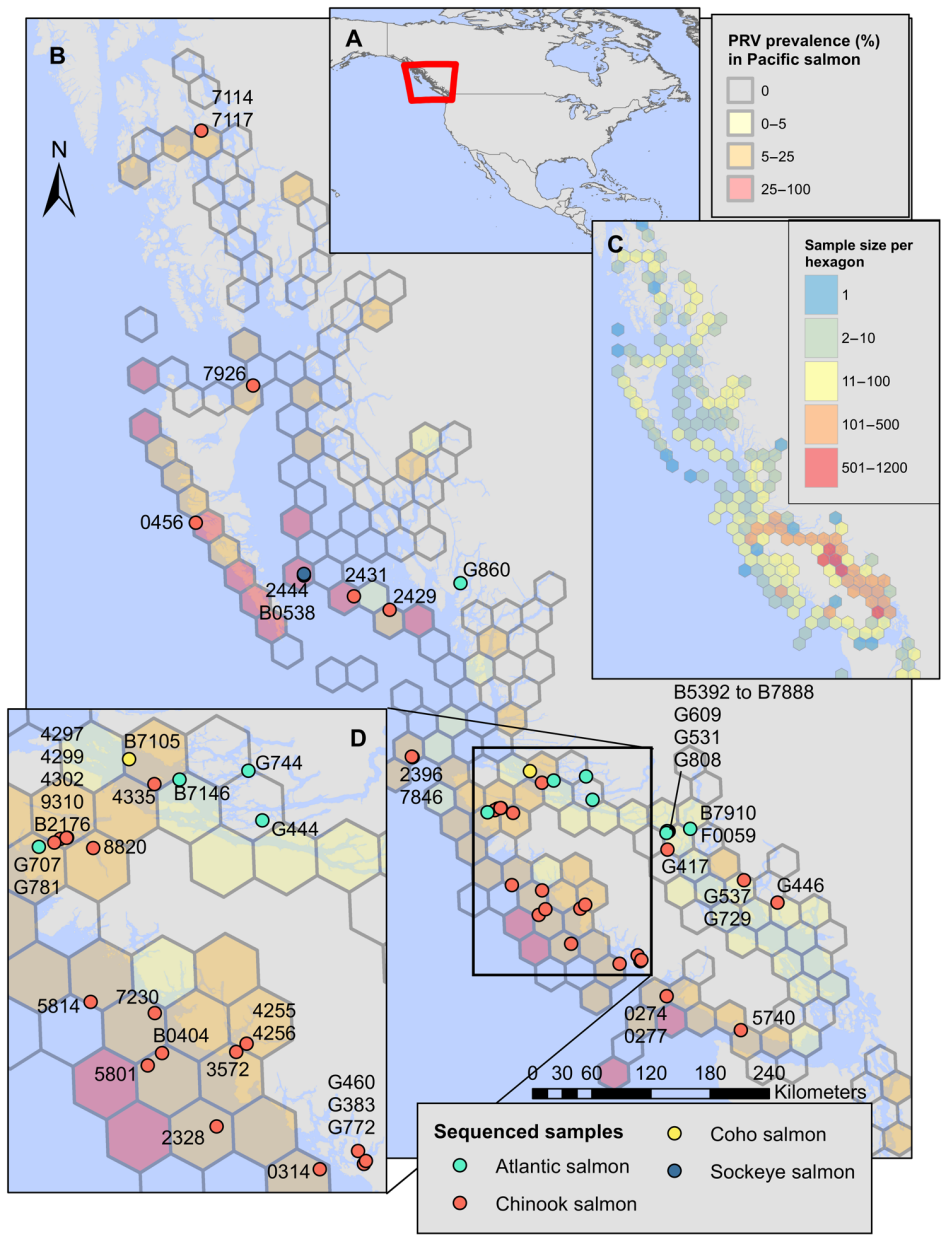
Epidemiological map of PRV-1 distribution and prevalence. Prevalence of PRV (hexagons) along the British Columbia coastline in three wild-sampled salmon species (Chinook, *n* = 6412; coho, *n* = 2132; sockeye, *n* = 3845) and position of sequenced aquaculture and wild samples (points). (**A**) Location of BC coast on map of North America. (**B**) PRV prevalence and sample sites along BC coastline. (**C**) Sample size of wild-sampled fish per hexagon. (**D**) Expanded region with high sequence sample density from (B).

Overall, PRV-1 prevalence was higher on the west coast of Vancouver Island (Fig. 3), where local populations of Chinook and coho salmon coexist in sheltered inlets with farmed salmon for up to their first year at sea (*54*). This is in contrast to the southern portion of the Salish Sea, on the east coast of Vancouver Island, where substantial Fraser River and east coast Vancouver Island salmon populations enter the ocean. Here, farms are absent, and farm exposure may primarily occur more fleetingly during northward migration (*22*).

### Transmission between populations

**Fig. 4:**
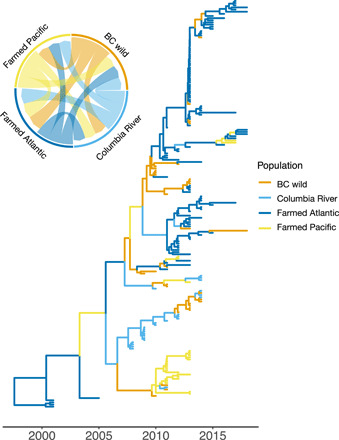
Phylogenetic evidence of transmission between populations. Maximum clade credibility phylogeographic tree of samples from the Eastern Pacific (*n* = 190) (based on full genome sequences where available). The tree is time scaled, and branch colors depict the most probable inferred population ancestor (populations originating from BC rivers and hatcheries, the Columbia River, farmed Atlantic salmon, and farmed Chinook salmon). The inset circular plot depicts the relative rates of transmission (depicted by the thickness of the arrows) between populations in the NE Pacific. These rates were estimated by the structured coalescent model implemented in BEAST 2.6.2. Note that phylogeographic reconstruction is sampling dependent, and in our study, the number of farmed (*n* = 133) and wild/hatchery (*n* = 57) salmon is relatively equal, whereas we estimate the number of PRV-infected farmed fish to vastly exceed the number of PRV-infected wild fish (table S3). Therefore, we predict that our model may substantially underestimate transmission from farmed to wild salmon.

We examined genomic data for evidence of transmission between farmed and wild salmon. The approach was similar to the interoceanic analysis (Fig. 1) but focused on NE Pacific samples. The placement of samples from four NE Pacific “populations” within our phylogenetic reconstruction yields information about PRV’s transmission history. A lack of PRV-1 transmission between these populations would have resulted in separate respective clades. Instead, we found wild and farmed salmon shared clades (Fig. 4), irrespective of our method of phylogenetic reconstruction or genomic segment (Fig. 1 and figs. S5 and S6). This corresponds with findings from Norway, where there was similar evidence for viral exchange between farmed and wild populations (*15*).

Despite sampling for six consecutive years, the majority (23 of 25) of PRV-1 sequences from Columbia River Chinook share the same PRV-1a clade (clade 1; figs. S5 and S7). Clade 1 did not contain any BC-farmed salmon, suggesting that Columbia River Chinook salmon are not the primary source of PRV transmission to BC’s farmed salmon, and evidence for sustained transmission of a unique lineage of PRV-1a within the Columbia River salmon. Because of the similarity in PRV-1 lineage within these fish and the high prevalence of PRV-1 within this population (fig. S4), we hypothesize that these fish became infected in freshwater hatcheries, where the same lineage of PRV-1 is sustained year upon year. Two Columbia River coho salmon, however, did harbor the lineage of PRV-1a found in farmed Atlantic salmon in BC (clade 2 in fig. S5), suggesting that marine infection in coastal BC can also occur.

We used multiple lines of evidence to infer direction of PRV-1 transmission. First, we reconstructed the ancestral source populations of sequences within the NE Pacific under the structured coalescent model (*46*), as implemented in BEAST 2.6.2 (Fig. 4). Our estimates of migration rates among the four NE Pacific populations support multiway transmission (Fig. 4 and table S2). Second, we looked for instances of paraphyly (viruses sampled from wild salmon nested with clades of viruses sampled from farmed salmon or vice versa) in our maximum likelihood (ML) phylogenies (figs. S5 and S6). We observed multiple instances of viruses sampled from wild salmon being paraphyletic within clades of viruses sampled from farmed. Together, these findings suggest transmission in both directions between farm and wild populations. An important caveat, however, is that phylogeographic reconstruction is sampling dependent (*55*), and in our study, the number of sequences was relatively balanced between farmed (*n* = 133) and wild (*n* = 57) salmon, with relatively few samples for such a wide temporal period. Another limitation is the low confidence in transmission events further back in time; however, these older and less confident events contribute to the estimated transmission rates with equal weight as more recent events, which are estimated with a higher confidence.

We also estimate that the number of PRV-infected farmed fish vastly exceeds the number of PRV-infected wild fish (table S3), and therefore, the model may substantially underestimate transmission from farmed to wild salmon. Furthermore, our results suggest that freshwater (e.g., Columbia River) hatcheries are a source of PRV transmission. However, transmission is likely higher from farmed net-pen than hatchery smolts, because mortality of ocean-going fish is comparatively high (*56*) relative to net-pen–reared fish. For instance, it has been documented that infectious hematopoietic necrosis virus (IHNV)–infected salmon smolts are more likely to be consumed by a predator (*57*), which would result in lower prevalence of infected fish in the wild. Nevertheless, our analysis provides important insight into transmission dynamics that would be impossible without genomic data. Our earlier lines of epidemiological evidence (Fig. 2) corroborate these phylodynamic results and further suggest that the predominant direction of transmission of PRV-1 is from farmed to wild.

### Historical population dynamics of PRV-1 in the NE Pacific

**Fig. 5:**
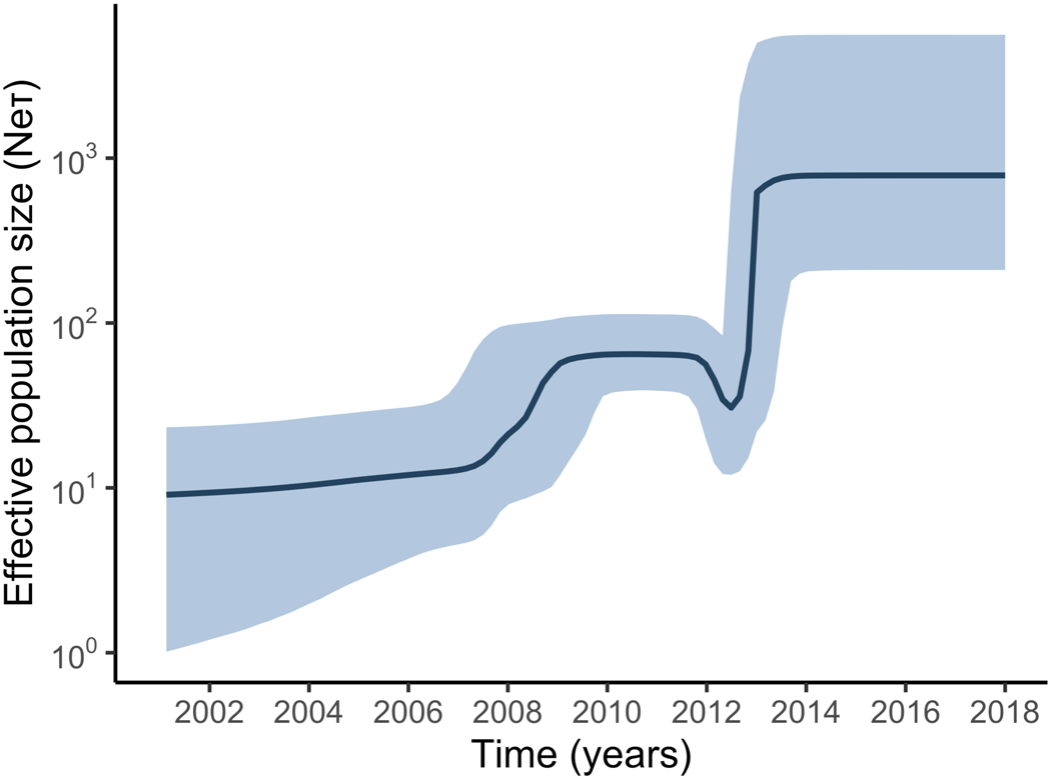
Phylogenetic assessment of PRV-1 population dynamics. Median estimated PRV-1 effective number of infections in the NE Pacific. The 95% highest probability density is shaded in light blue, and the black curve represents the median estimated effective number of infections through time (effective number of infections × generation time).

The shape of the time-scaled phylogeny (Fig. 4) contains information about the epidemiological dynamics of the virus population (*58*). PRV-1 was discovered relatively recently in BC, and there is a lack of reliable archival evidence of its historical presence in the region. Since its introduction to the NE Pacific, it is unclear whether PRV-1 has grown in population size (characteristic of an epidemic) or has been stable. We used a phylogenetic assessment restricted to samples collected in the NE Pacific to infer the historical demography of PRV-1a. PRV-1 population dynamics were reconstructed using Bayesian skyline plots (*46*) depicting the effective number of infections (*N*_e_) multiplied by the generation time (τ) through time (Fig. 5). The shape of the curve represents the number of infected fish that go on to infect additional fish over time. The analysis suggests an expansion of PRV-1 effective population size, with the largest increase occurring in the first half of the last decade (2010–2015), supporting the argument that PRV population size is not stable but rather has been expanding over time since the introduction of PRV to the NE Pacific. This is in contrast to IHNV, which has a constant population size (*59*). IHNV is thought to originate from the NE Pacific and therefore has a longer coevolutionary history with its host species and hence is more likely to display stability in host populations over time. The results of our demographic reconstruction represent an additional line of evidence supporting the hypothesis that PRV-1 arrived relatively recently in the NE Pacific (Fig. 1) and has since grown rapidly in population size. This pattern aligns with regional growth in aquaculture and associated prevalent PRV-1 infection in farms (Fig. 2A and fig. S3A). The expansion of PRV-1 is a concern considering poor documented returns of southern BC Chinook salmon in the same time frame (*4*, *60*).

The rise of aquaculture has caused an ecological shift that favors the emergence and spread of marine infectious disease. Our analyses provide multiple lines of evidence implicating aquaculture in the emergence of a viral pathogen at both global and local scales, highlighting the potential for the introduction of infectious agents into naive wild populations. Our study highlights the need for robust regulation of aquaculture that may prevent future losses in wild populations, which we propose may be exacerbated by PRV-1 and other emerging infectious diseases. Because of the logistical barriers to studying disease processes in wild fish, there have been few studies that have been able to quantitatively investigate the role of disease in population declines. However, because there is evidence that both wild and farmed Chinook are susceptible to a disease that appears to be caused by PRV-1 (*33*, *39*) and emerging viruses associated with farmed and hatchery salmon have been detected in wild Chinook (*13*, *20*), we believe that a precautionary approach is warranted.

We show that PRV-1 originates in Europe, and despite historical introductions of Atlantic salmon to the Pacific Ocean in the 19th century, we estimate that PRV-1 was introduced much more recently, since the expansion of aquaculture in the 1980s. Regionally, we show that, since PRV-1a’s introduction to the NE Pacific, the number of infections has increased, and this increase appears to be mediated by continual transmission between cultured and wild salmon. Surveillance and genome sequencing of PRV-1 provides evidence that salmon farms serve as a source of PRV-1 infection to wild salmon in the NE Pacific.

Our findings also suggest that PRV-1 could pose a risk to wild NE Pacific Chinook salmon. Mounting evidence suggests that, similar to other salmon species (*27*–*29*), Chinook salmon are susceptible to disease caused by PRV-1 (*33*, *39*). Thus, transmission of PRV-1 from farmed salmon to wild Chinook salmon and the consequent risk of disease need to be considered carefully in regulatory frameworks for aquaculture and for conserving wild salmon (e.g., the Federal Aquaculture Act, Species at Risk Act, and the Pacific Salmon Treaty).

The emergence of disease in agricultural systems remains a threat to food security, and aquaculture’s rapid domestication of marine species (*61*) has led to large farmed populations that result in increased density-dependent transmission and high risk of disease transfer in the marine environment (*62*, *63*). Although infectious disease is just one of many threats to marine organisms, fully understanding the health status of at-risk wild populations will only be possible if management agencies continue to invest in active monitoring for emerging infectious diseases.

## MATERIALS AND METHODS

### Sampling

Samples of aquaculture salmon were obtained from the Aquaculture Management Division, Fisheries and Oceans Canada (2011–2013) and from the Strategic Salmon Health Initiative longitudinal sampling of Mowi and Cermaq farms (2013–2015). Sampling of wild juvenile salmon was conducted by the Environmental Watch Program and Strategic Salmon Health Initiative in coordination with the Fisheries and Oceans High Seas Program and Strait of Georgia Salmon Program, as well as by the Hakai Institute. All samples were collected in BC, including Columbia River fish. Although hatchery samples can sometimes be identified by adipose fin clips, not all hatchery fish are marked, so in this study, we use the term “wild” to describe any fish that are not in a net-pen. Population of origin was determined by genetic stock identification (*64*).

### Sample processing and genome sequencing

We conducted high-throughput (HT) reverse transcription polymerase chain reaction (RT-PCR) on tissue samples collected from thousands of Chinook (*n* = 6791), coho (*n* = 2165), and sockeye (*n* = 4140) salmon collected in the marine environment in their first year of marine residence around Vancouver Island, BC (Fig. 3). RT-PCR surveillance of PRV-1 was carried out using the Fluidigm BioMark HT–quantitative PCR platform to quantify the presence and relative load of viral RNA. Sample preparation, RNA extraction and normalization, cDNA synthesis, specific target amplification, incorporation of artificial control standards and processing controls, and assay validation were completed according to previously described and validated protocols (*65*).

To enable full genome coverage while still multiplexing several samples at a time, we applied target enrichment using the SureSelect RNA Direct next-generation sequencing (NGS) target workflow (Agilent, Santa Clara, CA, USA). RNA sequencing libraries were prepared as previously described using the SureSelect Strand-Specific RNA library Prep kit (Agilent, Santa Clara, CA, USA) (*48*). We used a custom set of RNA target enrichment probes designed to genomes of known salmon infectious agents, which included representative PRV genomes. All NGS samples were processed on the v2 300 Illumina MiSeq sequencing platform.

Raw paired-end reads, 100 base pairs (bp) in length, were filtered using default Trimmomatic (*66*) settings to eliminate reads less than 36 bp in length, trimming adapters, trimming leading or trailing bases with Phred base quality (BQ) score <3, and excluding strings of four bases whose average per BQ score was <15. Resulting trimmed reads were assembled using a reference-guided alignment to a PRV-1 reference sequence (GenBank accession numbers NC_036468 to NC_036477) using the Burrows-Wheeler Aligner (BWA-MEM) algorithm (version 0.7.17) (*67*) with default parameters. We subsequently used SAMtools (version 1.9) (*68*) to eliminate unmapped reads, visually inspected alignments in the Geneious genome browser (version 10.2.6) (*69*), and generated majority consensus genome sequences. Genome sequences are available on GenBank (accession nos. MT758702-MT759571 and MT778887-MT778996).

### Phylogenetic analysis

Our analyses combined our newly assembled PRV genomes with all publicly available PRV-1 S1 sequences and PRV-1 full genome sequences (all S1 sequences were trimmed to the shortest minimum length, 731 nt). PRV-1 sequences were assembled by reference alignment using the MUSCLE alignment (*70*) plugin within Geneious (*69*). Model selection was carried out using MODELTEST (*71*) and the General Time Reversible (GTR) substitution model was selected. RAxML was used to construct all ML trees using the GTRCATI model, 100 distinct starting trees, and 1000 bootstraps (*72*). The tree was rooted according to the best-fitting root function within TempEst (*73*). We performed clock model selection as implemented in the PathSampling module in the BEAST 2.6.2 (*46*). Estimates of divergence and transmission events were calculated using the BEAST 2.6.2 and the MultiTypeTree package (*46*). We used the GTR substitution model under the assumption of a relaxed (exponential) clock model. To ensure that the results were robust to the clock model chosen, we also performed analyses under a relaxed (lognormal) clock model and found the divergence estimates were roughly concordant with both models. Trees were visualized and annotated within R using GGtree (*74*) and treeio (*75*). The ML tree (fig. S5) can be dynamically explored using the microreact (*76*) web server at https://microreact.org/project/Z_ogNOxVR.

The number of effective PRV-1 infections was calculated using a Bayesian skyline model on a reduced dataset originating from the NE Pacific. We retained samples that we could identify as originating from BC Rivers, the Columbia River, Atlantic salmon farms, or Chinook salmon farms (fig. S7). Samples were grouped according to the population of the fish, determined by genetic stock id, or information within papers and on GenBank. Samples of unclear population origin were removed, including coho salmon caught on the west coast of Vancouver Island and market samples.

To determine whether there is a temporal signal in the NE Pacific dataset, regression of root-to-tip distances was performed in TempEst (*73*) and an ML tree built using RAxML (*72*) as described above. In cases where there were identical sequences in multiple years, we retained the sequence with the earliest sampling date. Plots were made using ggplot2 (*77*).

### PRV epidemiological modeling

For the modeling of PRV prevalence in aquaculture (Fig. 2A and fig. S3A), we used a Bayesian binomial generalized linear mixed model with a logit link and flat priors to describe the probability that a given sample would be infected after a specified time in the ocean. Prediction trends were separated by genus, *Salmo* (*n* = 664) and *Oncorhynchus* (*n* = 182), and the model included random intercepts for sampling event (*n* = 252) and farm (*n* = 79) and random infection rates (i.e., slopes) for farm and hydrographic aquaculture management zones (*n* = 7).

The same modeling approach was used to describe the relationship between wild Chinook infection and distance to active aquaculture (Fig. 2, B and C). We tested the association of distance to aquaculture and PRV prevalence in Chinook, because they are the most abundant and stationary species over multiple seasons. Random intercepts included joint adaptive zones (*78*) and ocean entry year. Seaway distances from sampling locations of wild fish to nearest active net pens were estimated using the gdistance package in R (*79*). We divided the samples by season, fall-winter (*n* = 2279) and spring-summer (*n* = 2686) to account for seasonal differences in populations.

## SUPPLEMENTARY MATERIALS

Supplementary material for this article is available at http://advances.sciencemag.org/cgi/content/full/7/22/eabe2592/DC1
